# Comment on “Rivalry in *Bacillus subtilis* colonies: enemy or family?”

**DOI:** 10.1039/c9sm02141h

**Published:** 2020-03-24

**Authors:** Daniel Matoz-Fernandez, Sofia Arnaouteli, Michael Porter, Cait E. MacPhee, Nicola R. Stanley-Wall, Fordyce A. Davidson

**Affiliations:** Division of Molecular Microbiology, School of Life Sciences, University of Dundee Dundee DD1 5EH UK n.r.stanleywall@dundee.ac.uk; Division of Mathematics, School of Science and Engineering, University of Dundee Dundee DD1 4HN UK f.a.davidson@dundee.ac.uk; School of Physics, University of Edinburgh EH9 3FD UK

## Abstract

It is well known that biofilms are one of the most widespread forms of life on Earth, capable of colonising almost any environment from humans to metals.

It is well known that biofilms are one of the most widespread forms of life on Earth, capable of colonising almost any environment from humans to metals.^1^ In general, biofilms manifest as self-organised multicellular communities embedded in a self-produced extracellular polymeric matrix that, among other functions, aids signalling to the resident cells.^2^ For processes such as formation and dispersal, the importance of signalling at the cell-to-cell scale within the microbial community is widely recognised.^3,4^ However, in recent years it has been shown that bacteria in biofilms and other collectives of cells, can communicate effectively over large distances both within and between collectives using a diverse range of mechanisms including quorum sensing,^5^ electrical signalling,^6,7^ and mechanical transmissions.^8^

In a recent paper, Paul *et al.*^9^ have shown proximal *Bacillus subtilis* subspecies *spizizenii* biofilms interact, leading to either “demarcation” or “merging” of the initially spatially separated communities. Explicit definitions of demarcation and merging are not given, but it is inferred that the former implies that a visible gap is observed between two proximally located colonies and that this gap is a (semi-)permanent feature once established. The centre of this approximately linear gap is defined as the demarcation line (DL). Merging is the absence of this gap where proximal colonies grow until they visually appear to meet and importantly, the authors make explicit reference to “merging into a single colony”. The outcome of the interaction was found by Paul *et al.* to depend on the initial separation distance at the point of inoculation and the substrate composition. Moreover, by using an inert object (made from polydimethylsiloxane (PDMS)), in place one of the living communities, the authors concluded that these outcomes are driven by biochemical signals, not mechanical cues. The generality of these findings was supported by the demonstration that *Pseudomonas fluorescens* biofilms respond in a similar way. Finally, the authors explored various hypotheses to explain their experimental observations *via* mathematical modelling. These data shed significant light on the complexities of biofilm growth and interactions and provide impetus for future lines of research.

In this commentary, we support the inference that the outcome of an interaction between two neighbouring colonies is governed by long-range (and therefore most likely) biochemical signalling of some form. Moreover, we agree that the simple relationship presented between inoculum distance and diffusivity within the medium does capture the essence of the experimental observations. However, we argue that in general, the processes mediating the outcome of such interactions are far more complex than the authors postulate. In particular, we highlight that (i) their relationship does not necessarily hold in broader, but closely related contexts and (ii) the relationship does not elucidate the underlying mechanism(s).

With reference to the above, we found that if we establish two sibling *Bacillus subtilis* NCIB 3610 biofilms with an inoculation distance of 10 mm, at 48 hours we observed an apparent demarcation zone (Fig. 1a) similar to that presented in Paul *et al.* and reported by other authors cited in that paper (of particular relevance are ref. 10–12). However, we found that this demarcation zone resolved completely over time (Fig. 1b) and therefore does not represent a quantitatively predictable endpoint. Hence, we propose that evolution over realistic growth timescales has to be factored into future investigations. We also wish to highlight that confocal microscopy revealed that when two sibling biofilms appear to “merge” when imaged at the macro scale (*i.e.* there is no visible gap in between) they actually simply abut, and remain as distinct spatially segregated communities (Fig. 1c). Therefore, we suggest that “abuttal” rather than “merging” may be a more appropriate term, and argue against the concept of “merging into a single colony”, which implies mixing. Indeed abuttal and true merging may be distinct outcomes – this requires deeper investigation in each case.

**Fig. 1:**
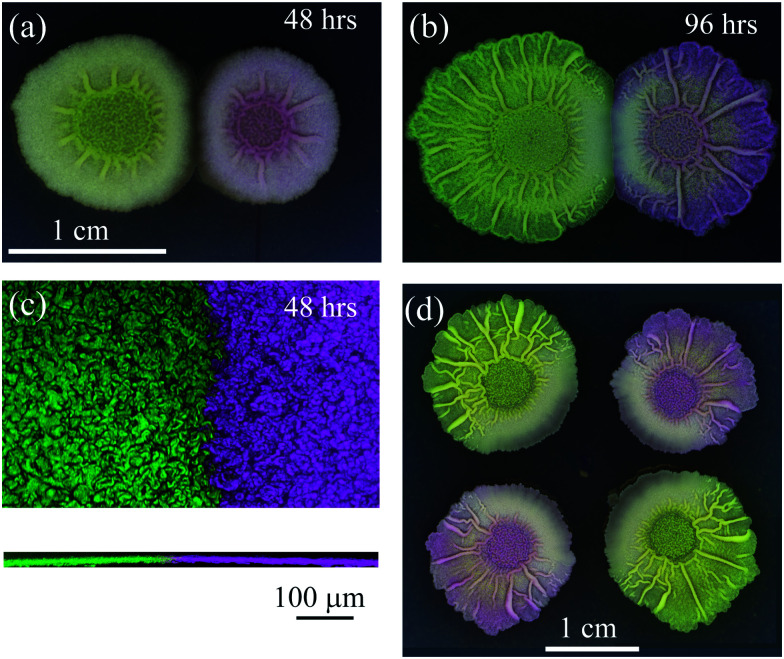
Interacting *Bacillus subtilis* biofilms. *B. subtilis* isolate NCIB 3610 was inoculated on 1.5% (w/v) agar MSgg medium at 30 °C.^14^ The otherwise isogenic strains were distinguished by expression of either mKate2 or gfp mut2. The founding cells of the biofilms were initially inoculated at a separation distance of 10 mm and imaged at (a) 48 h, where a distinctive demarcation zone is visible and indicated by a visible gap and resultant asymmetry of radial growth manifest as flattened edges along the interaction zone [expressing gfp – left and mKate2 – right]; and (b) at 96 hours, where the demarcation zone has resolved and the visible gap has disappeared. The biofilms now seem to “merge” as defined in Paul *et al.* (c) Merging of proximal biofilms using confocal microscopy maximum projection image for *x*–*y* plane (top panel) and *z*-plane (bottom panel) of interacting *B. subtilis* colonies [expressing gfp – left and mKate2 – right] at 48 hours, separated by an initial distance of 5 mm growing on 1.5% (w/v) agar MSgg medium at 30 °C. (d) *B. subtilis* sibling biofilms exhibit complex interactions. Four NCIB 3610 biofilm sibling colonies (two expressing gfp [top left and bottom right] and two expressing mKate2 [top right and bottom left]) were founded at 20 mm distance on 1.5% (w/v) agar MSgg medium at 30 °C and incubated for 96 h before imaging.

Finally, in our opinion, the mathematical model presented by Paul *et al.* does not support the conclusions of the paper. The paper proposes that biochemical signalling is responsible for controlling the interactions. This is in line with the work of Be’er *et al.*^12^ The model of Be’er *et al.* includes explicit production, sensing and reaction to a diffusible toxin and demarcation results as an emergent feature of the interaction of these fields. On the other hand, the model of Paul *et al.* includes no explicit reference to a biochemical signalling agent. Rather, the outcomes of their model are governed by a nutrient-dependent mortality rate (see eqn (8)) that is mediated by carefully selected time and space constraints. These temporal and geometric conditions are necessary to locate the DL at the interfacial gap between the colonies. These conditions therefore drive the conclusions of the model and hence, with respect and in our opinion, represent a circular argument. The authors are clear in their intention that the model should be general. It is our opinion that this generality precludes further understanding of the underlying mechanisms.

It could well be that biochemical signalling is exactly the mechanism that allows sibling colonies to determine both their temporal state and their relative spatial location. Investigating these hypotheses would be of great interest. We note however, that nutrient limitation in itself is sufficient to inhibit growth (see for example the models of ref. 10 and 11). In particular, in Arnaouteli *et al.*, 2019^13^ we demonstrated that proximal sibling colonies can have a marked effect on the growth dynamics of each colony where demarcation or abuttal was determined by nutrient limitation (see *e.g.* Fig. 6c^13^). The experimental results in Arnaouteli *et al.*, 2019 were rationalised using a mathematical model that took full account of the main components of the underlying hypotheses (in that case the production of pulcherriminic acid to chelate free iron in the medium). Growth arrest was an emergent property of this model. Of particular interest is that growth arrest of the expanding outer colony edge was observed in single biofilms – no interactions were necessary (although interactions were considered). The mathematical model elucidated this phenomenon to be induced by a wave of iron depletion that overtook the expanding colony margin. We therefore propose that when studying colony interactions, self-limitation is a feature that cannot be dismissed without further investigation.

We fully agree with the authors that proximal growth can have a distinct effect on the physical appearance of biofilms at the macroscale. For example, when we inoculated four sibling biofilms at sufficient distance to preclude abutting, a marked asymmetry of radial growth and of material properties as indicated by changes in wrinkle structure was still observed (Fig. 1d, see also ref. 11). This is suggestive of long-range (biochemical) signal(s) being transmitted and received at the cellular level and manifest through macro-scale remodelling of the biofilm architecture. However, our previous work and the experiments detailed here, lead us to conclude that in general the interaction between proximal biofilms is mediated by a complex growth response. This response can be mediated by separation and substrate stiffness, but is also dependent on multiple other factors including inoculum density and size, genus, media composition and other experimental constraints.

In conclusion, there is clear evidence that bacterial biofilms interact over short and long distances and that these interactions are in part governed by biochemical signalling. Moreover, truly understanding how microbes colonise and interact with their environment requires the scientific investigation of multi-biofilm assays. The physics/mathematics of soft matter communities are likely to have a significant input to future scientific studies in this area and contributions will best be made by careful matching of experimental data and biological understanding with mathematical models. This calibrated interdisciplinary approach is well-suited to shine further light on the fundamental role of microbes in the environment.

## Methods

### Biofilm formation

*B. subtilis* NCIB 3610 was grown on MSgg medium (5 mM potassium phosphate and 100 mM MOPS at pH 7.0 supplemented with 2 mM MgCl_2_, 700 μM CaCl_2_, 50 μM MnCl_2_, 1 μM ZnCl_2_, 2 μM thiamine, 0.5% glycerol, 0.5% glutamate) solidified with 1.5% select agar (Invitrogen) at 30 °C for the indicated time points as previously described^14^ with the exception that 1 μl of culture was used for biofilm initiation. Images of colony biofilms were recorded using a Nikon D3200 digital camera mounted on a Kaiser RS3XA copy stand or using a Leica MZ16FA stereomicroscope.

### Confocal microscopy

4 ml of MSgg medium supplemented with 1.5% (w/v) agar was placed into a 35 mm diameter Petri dish and dried for 1 hour in a laminar flow hood. NCIB 3610 strain constitutively producing GFP-^16^ or mKate2^17^ was spotted into the centre of the agar in the Petri dish and incubated at 30 °C for the indicated time period. A Leica SP8 upright confocal was used to image the edge of the biofilm using a 10× 0.3 N.A. air objective and a heated chamber that was pre-warmed to 30 °C. A cling film tent was draped from around the objective and tucked loosely under the stage to eliminate airflow across the plate and minimise dehydration (and therefore shrinkage) of the agar. An additional 35 mm diameter Petri dish was filled with water and placed next to the biofilm plate to increase the humidity inside the tent. An argon-ion laser was used to excite the GFP at 488 nm and 2% power. Z-stacks capturing the full height of the biofilm border were specified based on the presence of GFP-containing cells and planes of 1024 × 1024 pixels were acquired quickly using a resonant mirror, averaging 16 scans per line. Images were imported into an OMERO^15^ server and figures were prepared using OMERO figure (http://figure.openmicroscopy.org/).

## Conflicts of interest

There are no conflicts to declare.

## Supplementary Material
